# Comparative Evaluation of Carbohydrate, Amino Acid, and Ionic Liquid Excipients for Flavivirus Vaccine Stabilization

**DOI:** 10.1021/acsomega.6c00106

**Published:** 2026-03-12

**Authors:** Muhammadiqboli Musozoda, Lauren M. Paul, Chayah A. Boyd, Zachary J. Metott, David S.-J. Jang, Patrick C. Hillesheim, Scott F. Michael, Arsalan Mirjafari

**Affiliations:** † Department of Chemistry, 14828 State University of New York at Oswego, Oswego, New York 13126, United States; ‡ Department of Biological Sciences, 3391 Florida Gulf Coast University, Fort Myers, Florida 33965, United States; § Department of Chemistry, 6049 Illinois State University, Normal, Illinois 61761, United States

## Abstract

Live-attenuated flavivirus vaccines (yellow fever, dengue, and Japanese encephalitis) exhibit poor thermal stability in liquid formulations, requiring lyophilization and storage between 2 and 8 °C to maintain potency. We evaluated carbohydrates, amino acids, and choline-based ionic liquids as preservatives for vaccine-like flavivirus. Among the carbohydrates tested, trehalose and sucrose provided greatest stabilization, while histidine demonstrated the strongest stabilizing effect among amino acids. Trehalose-histidine and sucrose-histidine combinations produced synergistic effects, preserving viral infectivity more effectively than individual components. Choline chloride and choline acetate formulations, despite their established efficacy in protein stabilization, demonstrated limited enhancement of flavivirus thermal stability compared to carbohydrate-amino acid formulations. Trehalose-histidine combinations provided up to 19.4-fold improved titer retention compared to buffer controls, with consistent superiority across all three flavivirus species tested, while choline-based ionic liquid formulations showed moderate stabilizing effects but were consistently outperformed by carbohydrate-amino acid approaches.

## Graphical abstract

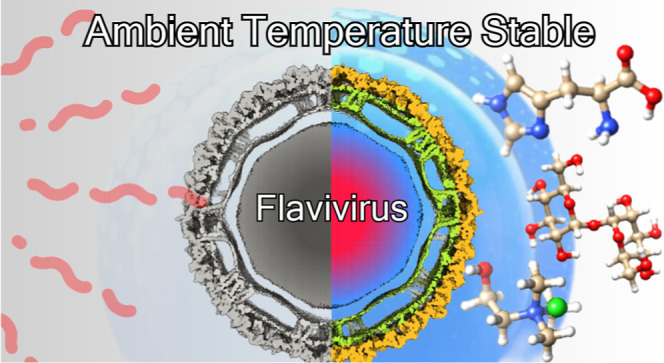

## Introduction

Nearly all licensed vaccines require uninterrupted refrigerated supply chain to maintain potency, yet cold chain failures are widespread and economically burdensome, particularly in resource-limited settings where maintenance consumes the majority of vaccination program costs.^1–6^ These global challenges are particularly pronounced for live-attenuated flavivirus vaccines—yellow fever (YFV), dengue (DENV), and Japanese encephalitis (JEV) where cold chain failures directly impact disease control in tropical regions most affected by these mosquito-transmitted viruses. Yellow fever affects ∼200,000 individuals annually with 30,000 deaths, primarily in Africa,^7,8^ while dengue has become the fastest-growing mosquito-borne disease with over 14 million cases in 2024 alone (a historic high that represents a 2-fold increase compared to 2023).^9^ Japanese encephalitis causes 100,000 clinical cases annually across 24 endemic countries, with case fatality rates reaching 30%.^10^ Collectively, over 900 million people live in flavivirus-endemic areas where cold chain limitations restrict vaccine access, yet no specific antiviral treatments exist, making prevention central.

Current live-attenuated flavivirus vaccines face unique stability challenges due to their complex multicomponent structure. These vaccines containviral proteins, nucleic acids, and lipid envelopes (Figure 1), making them susceptible to multiple degradation pathways. The flavivirus envelope glycoprotein is arranged in a dimeric herringbone pattern and is responsible for cell binding and membrane fusion through pH-triggered conformational changes involving specific histidine residues.^11–13^ Since premature pH-induced conformational changes render viruses inactive, flavivirus vaccines are particularly sensitive to buffering conditions and require stabilizing excipients that maintain optimal microenvironments around viral particles. Current flavivirus vaccines are lyophilized but still require storage at 2–8 °C, with yellow fever vaccine showing poor postreconstitution stability, requiring disposal after 1 h (Table 1).^14^

**Figure 1:**
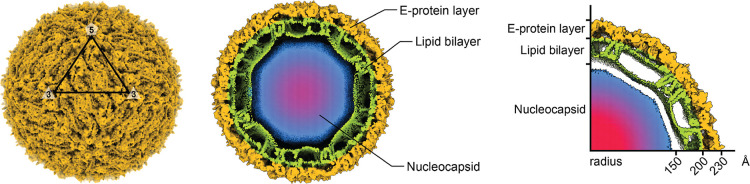
Cryo-electron microscopy structure of DENV-2 at 4 Å resolution. Left: surface representation showing icosahedral symmetry with 3-fold and 5-fold symmetry axes marked. Center and right: radial cross sections revealing the layered viral architecture: outer E-protein shell (gold), host-derived lipid bilayer envelope (green), and inner nucleocapsid core containing the viral RNA genome (blue-red gradient). The particle radius ranges from ∼150 to 230 Å, with total particle diameter of ∼500 Å.

**Table 1:** Thermal Stability Profiles and Storage Requirements of Currently Licensed Flavivirus Vaccines^a^

					thermal stability			
vaccine	excipients	formulation	freeze sensitive	storage temperature	2–8 °C	25 °C	37 °C	>40 °C
CYD-TDV/dengvaxia (DENV)	essential amino acids, nonessential amino acids, l-arginine HCl, sucrose, d-trehalose dihydrate, D-sorbitol, trometamol, urea	lyophilized	yes	2–8 °C	no data	no data	no data	no data
TAK-003/qdenga (DENV)	trehalose dihydrate, poloxamer 407, HSA, potassium dihydrogen phosphate, disodium hydrogen phosphate, potassium chloride, sodium chloride	lyophilized	yes	2–8 °C	24 months	no data	no data	no data
YF-VAX (YFV)	sorbitol, gelatin	lyophilized	yes	2–8 °C	no data	no data	*t*_1/2_ = 14 days	*t*_1/2_ = 3–4 days
IMOJEV (JEV)	mannitol, histidine, glutamic acid, lactose, potassium hydroxide, HSA	lyophilized	yes	2–8 °C	36 months (estimate)	no data	no data	no data
SA14-14-2 (JEV)	gelatin, sucrose, lactose, carbamide, HSA, BSA	lyophilized	no	2–8 °C	1.5 years	4 months	7–10 days	no data

a All vaccines are lyophilized formulations of live-attenuated virus with standard storage requirements of 2–8 °C. Excipient abbreviations are defined as follows, human serum albumin (HSA) and bovine serum albumin (BSA).

Excipients operate through distinct mechanisms to address these challenges. Carbohydrates form rigid amorphous matrices during dehydration (glass transition theory) that immobilize vaccine components and prevent degradative molecular motion.^15–17^ Preferential hydration represents a complementary mechanism where stabilizing agents like sugars and amino acids create protective water shells around viral proteins, maintaining native structure and increasing thermal resistance.^18^ While water replacement theory suggests direct sugar-protein hydrogen bonding in dried states,^19^ current evidence demonstrates that preferential hydration effects predominate in solution.^20^

Systematic evaluation of excipient combinations based on these established stabilization mechanisms is essential for developing optimized formulations. Nevertheless, limited studies have examined these mechanisms specifically for live-attenuated flavivirus vaccines, where lipid envelope integrity presents unique stabilization challenges distinct from other vaccine types.

Our preliminary *in silico* studies demonstrated that trehalose and choline chloride effectively stabilize nucleic acids under thermal stress,^21^ aligning with experimental work by Wiggan et al. showing trehalose-containing formulations enhanced YFV and DENV thermal stability with minimal titer loss after 8 h at 37 °C.^22^

To evaluate stabilizer options for flavivirus vaccines, we investigated both conventional approaches (carbohydrates and amino acids) and alternative choline-based ionic liquid (IL) formulations (Figure 2). We selected choline-based salts with three different anions (Cl^–^, AcO^–^, H_2_PO_4_^–^) because their counterions demonstrate protein-stabilizing properties through hydrogen bonding and electrostatic interactions, while choline represents a biocompatible, GRAS (Generally Recognized as Safe)-approved cation (Figure 2).^23–25^ This study represents the first comprehensive systematic evaluation of carbohydrates, amino acids, and choline-based ILs for flavivirus vaccine thermostability, using inexpensive, FDA-approved compounds. Our research demonstrates that these excipients can thermally stabilize DENV-2, YFV, and JEV vaccine models for 24 h at 21 °C under ambient conditions encountered in regions with unreliable cold chain infrastructure.

## Experimental Section

### Virus Models

DENV Strain New Guinea-2 (DENV-2) was provided by Robert Tesh at the University of Texas at Galveston through the World Reference Center for Emerging Viruses and Arboviruses. Yellow fever virus (17D-YFV) is a molecular clone, pACNR-17D, provided by Charles Rice at the Rockefeller Institute.^26^ Japanese encephalitis virus (JEV) is a ChimeriVax, yellow fever virus construct with JEV SA14-14-2 premembrane and envelope protein substitution, provided by Tom Monath at Acambis.^27^

### Host/Target Cells

Vero African green monkey kidney cells from the American Type Culture Collection (Vero CCL-81, ATCC, Manassas, VA) were used as host/target cells for DENV-2, YFV-17D, and JEV in plaque forming unit titer assays. All cells were grown in Eagle’s Minimum Essential Medium (EMEM; Cat. 30-2003, ATCC) supplemented with 10% (v/v) fetal bovine serum (HI FBS; Gibco, Thermo Fisher Scientific, Inc., Waltham, MA), 2 mM Glutamax (GlutaMAX; Cat. 35050, Gibco, Thermo Fisher Scientific, Inc.), 100 U·ml^–1^ Penicillin G and 100 μg·mL^–1^ Streptomycin (Pen Strep; Gibco, Cat. 15140-122, Thermo Fisher Scientific, Inc.), and 0.25 μg·mL^–1^ amphotericin B (Amphotericin B; Gibco, Cat. 15290-018, Thermo Fisher Scientific, Inc.) at 37 °C with 5% (v/v) CO_2_.

### Plaque Forming Unit Assays and Titer Calculation

Vero African green monkey kidney target cells were seeded in 12-well or 24-well plates, 24 h prior to virus inoculation. Approximately 2 μL of virus, either DENV-2 (3.5 × 10^7^ PFU·mL^–1^), YFV-17D (8 × 10^7^ PFU·mL^–1^) or JEV (2 × 10^6^ PFU·mL^–1^), and 2 μL excipient solutions were combined and desiccated for 2 h at room temperature (21 °C) in a desiccator (Bel Art Space Saver Vacuum Desiccator, Bel Art SP Scienceware, Wayne, NJ) under vacuum pressure (approximately 18–19 in Hg) created by a diaphragm vacuum pump (PILOT3000, Lab Depot Inc., Dawsonville, GA) to remove moisture from solutions, using at least 40 g of indicator Drierite desiccant (W.A. Hammond Drierite Co., LTD, Xenia, OH). Once dried, samples were sealed in sterile O-ring, screw-cap tubes (Fisherbrand, Cat.02-681-372, Thermo Fisher Scientific Inc.) and incubated at room temperature for 24 h prior to inoculation. After 24 h of incubation, virus treated mixtures were resuspended in serum-free EMEM for 5 min and then serially diluted using serum-free EMEM. The virus mixtures were allowed to infect confluent target cell monolayers for 1 h at 37 °C with 5% (v/v) CO_2_, rocking every 10–15 min to distribute. The inoculum was aspirated and overlaid with 2X Modified Eagle's Medium (MEM) (MEM; Gibco, Cat. 11-935-046, Thermo Fisher Scientific, Inc.) supplemented with 10% (v/v) fetal bovine serum, 2 mM Glutamax, 100 U·mL^–1^ Penicillin G and 100 μg·mL^–1^ Streptomycin, 0.25 μg·mL^–1^ amphotericin B and microcrystalline cellulose Avicel solution 1.2% (w/v) (FMC BioPolymer, Philadelphia, PA). The infected cells were incubated at 37 °C with 5% (v/v) CO_2_ for 72 h. After which the overlay was aspirated and infected cultures were fixed with 10% (w/v) formalin (Formalde-Fresh Solution, Buffered, Fisher Chemical, Cat. SF93-4, Thermo Fisher Scientific, Inc.) overnight at 4 °C, permeabilized with 70% (w/v) pure ethanol for 20 min minimum, and rinsed with 1X phosphate buffered saline solution, pH 7.4 (PBS, Fisher BioReagents Cat. BP665-1, Thermo Fisher Scientific) prior to immunostaining. The virus foci were immunostained using human monoclonal antibodies (hMAbs) 1.6D and D11C as previously described^28^ as primary tags in a 1X PBS solution with 0.1% (v/v) Tween 20 (Tween 20; Cat. 156054, Thermo Fisher Scientific Inc.), 5% (w/v) nonfat dry milk, and final antibody concentration of 1 μg/mL,^28^ rocked overnight at room temperature. A secondary antibody solution with 0.2% (v/v) concentration of horseradish peroxidase (HRP) conjugated goat anti-human IgG (H + L)­(Pierce, Rockford, IL) prepared in a 1X PBS solution containing 0.1% (v/v) Tween 20 was rocked overnight at room temperature. Foci were developed and visualized using 3,3-diaminobenzidine tetrahydrochloride (D5905; Sigma-Aldrich, St. Louis, MO) and 8 μL of 30% (v/v) hydrogen peroxide (H_2_O_2_) per 20 mL of 1X PBS. Wells with plaque forming units between 20–200 foci were recorded and used to calculate resulting titers for each treatment. Independent assays were performed at least twice for each treatment, with technical replicates (triplicate) performed within each assay. Titers from each biological and technical replicate were normalized using control average titers, such that stability could be compared across multiple, different treatment assays. Statistical analyses were performed using GraphPad Prism Software.

### Preparation of pH Buffer Solutions

The HEPES (4-(2-hydroxyethyl)-1-piperazineethanesulfonic acid, Cat. 091588413, MP Biomedicals Inc., Solon, OH) buffer solution was made at a 1 M final concentration in sterile, serum-free EMEM. The pH was adjusted to 7.4 with NaOH in deionized water while monitoring with Fisherbrand accumet pH meter (Thermo Fisher Scientific). The buffer solution was filter sterilized using a 0.22 μm pore size PES membrane (EZFlow; Cat. 371–2215-OEM, Foxx Life Sciences, Londonderry, NH). The buffer solution was stored at 4 °C when not in use.

A 10X PBS Buffer pH 7.4 (Ambion, Cat. P/N AM9625, Thermo Fisher Scientific Inc.) was diluted to 2X using ultrapure distilled water (Invitrogen, Cat. 10977-015, Thermo Fisher Inc.) before being combined with the virus during assays to reach a final concentration of 1X PBS. 1X concentration is equivalent to 0.01 M. Solution was stored at 4 °C when not in use.

Tris Base (Fisher BioReagents, Cat. BP152-1, Thermo Fisher Inc.) was measured out and resuspended in serum-free EMEM media at approximately 10% (w/v) and adjusted pH to 7 using NaOH as previously described above. The actual concentration of the solution was 0.765 M. The buffer solution was filter sterilized using a 0.22 μm pore size PES membrane (Foxx Life Sciences). The buffer solution was stored at 4 °C when not in use.

### Preparation of Excipient Formulations

Formulation concentrations were based on established vaccine stabilization protocols and preliminary optimization studies demonstrating optimal protective ratios for carbohydrate-amino acid combinations.^22,25^ Standardized formulations were prepared in several categories: individual components, combination formulations, and IL-based systems. Individual component formulations contained either 500 mg of carbohydrates (trehalose, sucrose, mannitol, dextrose, sorbitol, or pullulan) or amino acid components (340 mg l-arginine, 5 mg l-histidine, 22 mg l-histidine HCl, or 27 mg glycine) as single excipients. Combination formulations contained 500 mg carbohydrates with amino acid components (either individual sugars paired with individual amino acids, or 400 mg trehalose plus 100 mg pullulan combined with amino acids). IL-based formulations contained 1.0 g choline-based IL either alone or combined with carbohydrates and/or amino acids to create deep eutectic solvent (DES) systems.^25^ Individual components were weighed using an analytical balance (precision ±0.1 mg), with carbohydrates and amino acids premixed before ionic liquid addition for DES formulations. All formulations were resuspended in 1 M HEPES in serum-free EMEM at approximately 10% (w/v). Solutions were filter sterilized using a 0.22 μm pore size PES membrane (Foxx Life Sciences) and stored at 4 °C until use.

## Results and Discussion

### Optimization of Buffering Conditions

Forced degradation studies evaluate formulation stability by exposing samples to controlled stress conditions, enabling researchers to predict long-term degradation patterns and optimize excipient selection for enhanced stability and extended shelf life. pH manipulation represents one of the primary degradation pathways for protein-based formulations.^1,3^ Previous work has established that monoclonal antibodies exhibit increased aggregation tendencies at low pH values, driven by changes in hydrophobicity and net charge that determine protein-specific aggregation behavior across different pH ranges.^24,29^ This pH sensitivity proves particularly critical for flavivirus vaccines, where viral infectivity depends on pH-controlled endocytic processes that facilitate envelope-membrane fusion during cellular entry.^30^

Given the importance of pH stability in flavivirus vaccine formulations, we conducted a comparative study of three buffer systems: 4-(2-hydroxyethyl)-1-piperazineethanesulfonic acid (HEPES), 2-amino-2-hydroxymethylpropane-1,3-diol (Tris, trometamol), and phosphate-buffered saline (PBS) to evaluate their pH buffering effects in vaccine formulations. HEPES demonstrated superior stabilizing properties compared to other buffer systems (Figure 3). Addition of HEPES significantly improved the thermal stability of DENV-2 compared to formulations without buffering, with similar stabilizing effects observed for YFV and JEV, as shown in Figure 9. This finding aligns with current vaccine formulation practices, as the Smallpox/Mpox vaccine ACAM2000 includes HEPES as a stabilizing excipient.

**Figure 2:**
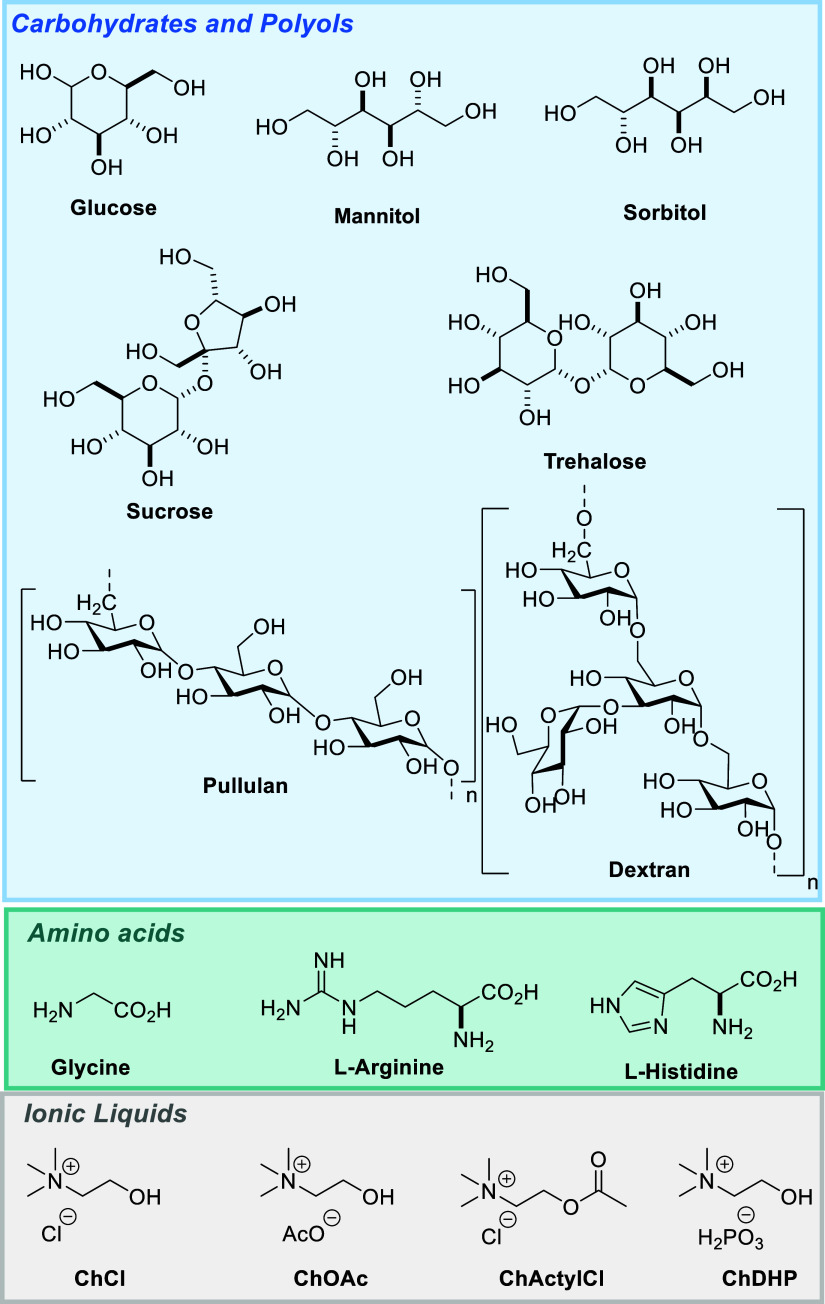
Chemical structures of carbohydrates, polyols, amino acids, and choline-based salts evaluated as excipients in this study.

**Figure 3:**
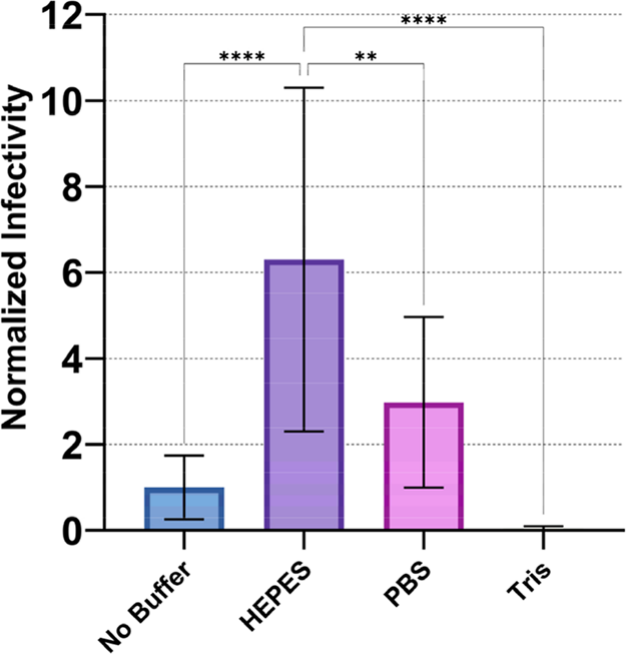
Thermal stability of DENV-2 NGC after 24 h incubation at 21 °C with and without buffer treatment. Error bars represent standard deviation of sample titers. Independent assays were repeated twice, minimally, with multiple technical replicates per assay. The resulting virus titers were normalized to the total average titer of DENV-2 without buffer treatment, represented as baseline for comparison, stability value of (1) in the graph. Normalized data were used to determine statistical significance with One-Way ANOVA test, Tukey’s HSD post hoc applied. Statistical significance is indicated as follows: *p < 0.05, **p < 0.01, ***p < 0.001, ****p < 0.0001.

In contrast, phosphate-buffered systems present well-documented disadvantages, particularly during freeze-thaw processes where crystallization and pH shifts can occur. Studies examining protein denaturation during freeze-thaw cycles in sodium phosphate buffered systems have demonstrated greater activity loss and structural changes compared to alternative pH buffers.^31^ An explanation of the difference in stability seen between HEPES and phosphate buffering systems could be due to the differential solubility of the monocation versus dication versions of the phosphate ion. During a drying (or freezing) process, less soluble disodium phosphate will precipitate first, causing a large change in pH.^31^ This result alone suggests that phosphate-based pH buffer systems are not preferable for biological drying or freezing applications, especially for pH-sensitive products like live-attenuated flavivirus vaccines (Figure 3). An explanation of the poor stability provided by Tris is less straightforward. Tris is used as a pH buffering system in several live-attenuated vaccines and has been known to stabilize flavivirus particles during cryo-electron microscopy imaging.^32^ Since Tris is also well-known to be toxic in cell cultures, we speculate that our results may be related to reduced cellular metabolism, although we did not observe any overt cytopathic signs in any of the experiments.

### Individual Carbohydrate and Amino Acid Effects

We initially evaluated Individual carbohydrates and amino acids to assess their effects on DENV-2 viral stability, infectivity, and potency (Figure 4). All tested carbohydrates demonstrated superior thermal stability compared to the HEPES control (Figure 4A). Sucrose and trehalose provided the highest stabilizing effect, followed by mannitol. Glucose, pullulan, and sorbitol showed lower stabilizing capacity, with glucose exhibiting the poorest performance among the carbohydrates tested when comparing averages. All carbohydrates tested were statistically significant in their stabilizing ability compared to the HEPES control but were not statistically different from each other except for glucose.

**Figure 4:**
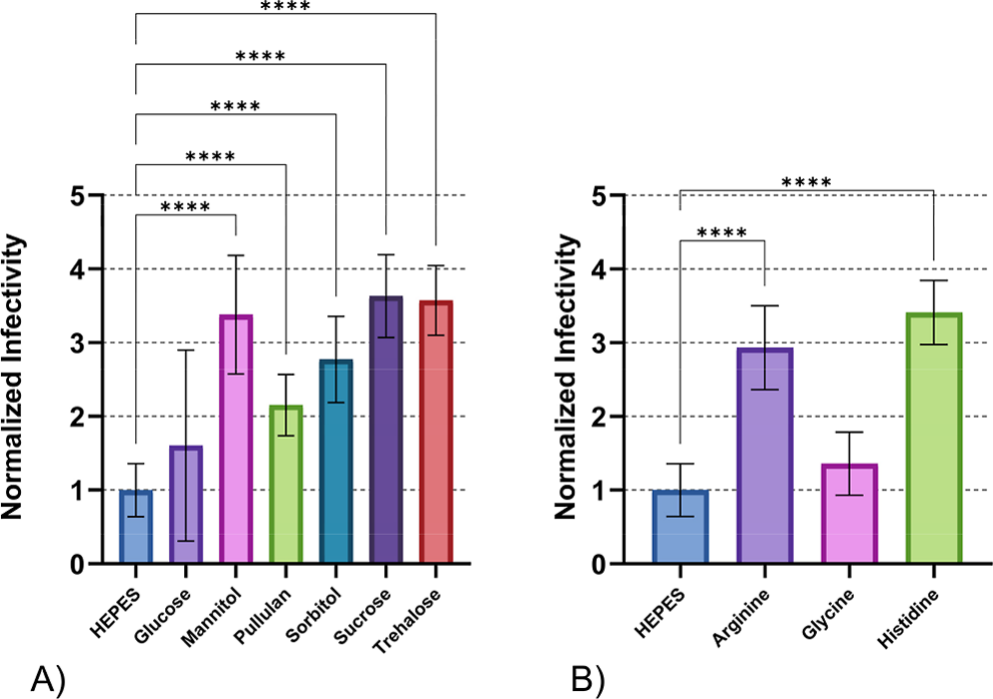
Thermal stability of DENV-2 after 24 h incubation at 21 °C for (A) individual carbohydrates and (B) individual amino acids. Error bars represent standard deviation of sample titers. Independent assays were repeated twice for each treatment, minimally, with multiple technical replicates per assay. Data were normalized to the mean viral titer of DENV-2 samples treated with HEPES buffer, represented as a stability value of (1) on the graph. Normalized data were used to determine statistical significance with One-Way ANOVA test, Tukey’s HSD post hoc applied. Statistical significance is indicated as follows: *p < 0.05, **p < 0.01, ***p < 0.001, ****p < 0.0001.

These findings align with previous research on H1N1 influenza subunit vaccines, where 4% trehalose and 4% sucrose were the top performers, while mannitol caused complete potency loss.^33^ The stabilizing effects of trehalose and sucrose in solution can be explained through preferential hydration and osmotic stabilization mechanisms.^34^ Trehalose and sucrose preferentially interact with water molecules rather than directly binding to viral proteins, creating organized hydration shells that maintain native protein conformations and resist thermal denaturation. Additionally, these disaccharides increase solution viscosity and osmolarity, which reduces molecular mobility and slows degradative processes. The anchorage hypothesis^35^ suggests that sugars interact with protein surfaces through residual water molecule networks, providing additional stabilization without disrupting native protein structure.^36^ Under our experimental conditions with brief drying, these solution-phase mechanisms predominate rather than the vitrification effects observed in fully lyophilized systems.

While both trehalose and sucrose provide stabilization through preferential hydration mechanisms, trehalose’s nonreducing nature and superior kosmotropic properties result in more effective hydration shell formation around viral proteins. This enhanced molecular organization provides better preservation of envelope protein conformations critical for maintaining viral infectivity under thermal stress conditions.

We individually tested three amino acids for their stabilizing effects on DENV-2. Histidine and arginine provided statistically significant stability compared to HEPES only control, respectively, while glycine demonstrated minimal stabilizing capacity (Figure 4B). Histidine is commonly used in vaccine formulations due to its dual role in stability enhancement and pH buffering.^37,38^ The stabilizing mechanism involves histidine shielding solvent-exposed hydrophobic regions on protein surfaces.^39^ These results correlate with the present findings where glycine provided the least protection.

### Combined Carbohydrate and Amino Acid Formulations

We evaluated combination formulations containing both carbohydrates and amino acids for potential synergistic stabilizing effects. Figure 5 presents these combination data organized by amino acid type, while Figure 6 groups the same formulations by carbohydrate category. Statistical analysis revealed that all combination formulations demonstrated enhanced thermal stability compared to the HEPES buffer control, with the sole exception of the sucrose–arginine combination.

**Figure 5:**
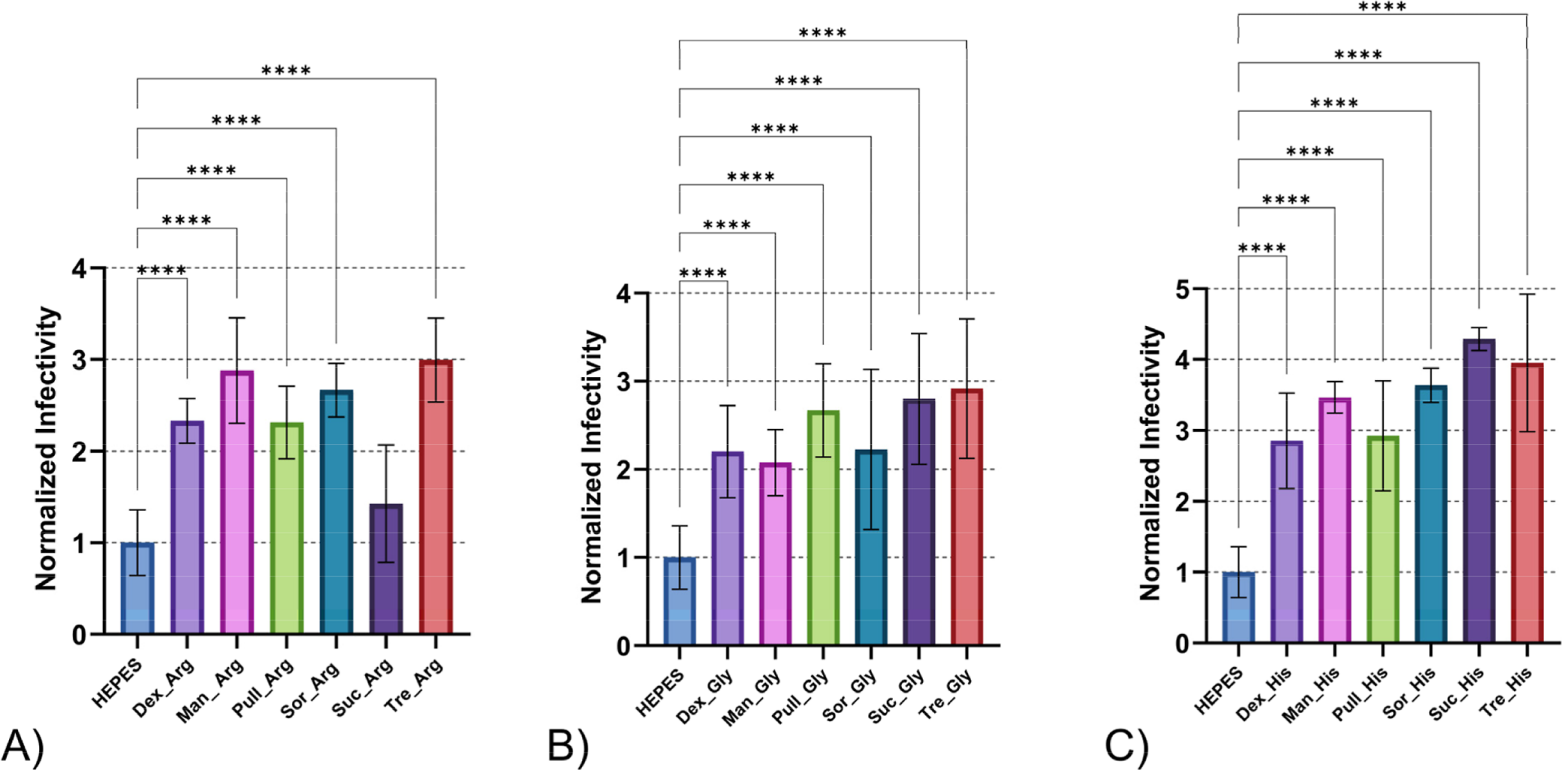
Thermal stability of DENV-2 after 24 h incubation at 21 °C for formulations with carbohydrates and amino acids grouped by amino acids: (A) carbohydrates and arginine formulations, (B) carbohydrates and glycine formulations and (C) carbohydrates and histidine formulations. Abbreviations: dextran (Dex), mannitol (Man), pullulan (Pull), sucrose (Suc), sorbitol (Sor), trehalose (Tre), arginine (Arg), glycine (Gly), histidine (His). Error bars represent standard deviation of sample titers. Independent assays were repeated twice for each treatment, minimally, with multiple technical replicates per assay. Data were normalized to the mean viral titer of DENV-2 samples treated with HEPES buffer, represented as a stability value of (1) on the graph. Normalized data were used to determine statistical significance with One-Way ANOVA test, Tukey’s HSD post hoc applied. Statistical significance is indicated as follows: *p < 0.05, **p < 0.01, ***p < 0.001, ****p < 0.0001.

**Figure 6:**
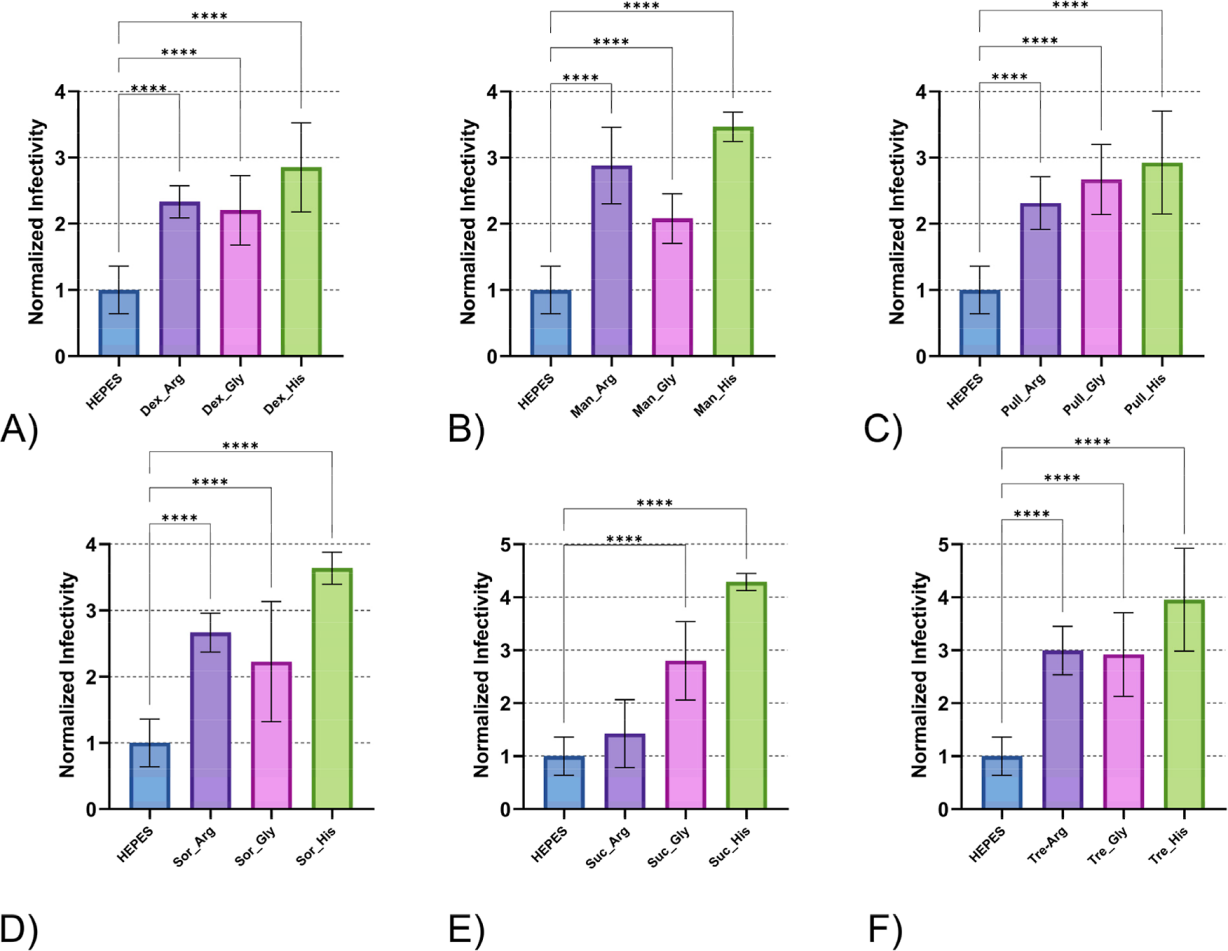
Thermal stability of DENV-2 after 24 h incubation at 21 °C for formulations with carbohydrates and amino acids grouped by carbohydrates. Panels show formulations containing (A) dextran, (B) mannitol, (C) pullulan, (D) sorbitol, (E) sucrose, and (F) trehalose, each combined with the three amino acids. Abbreviations: dextran (Dex), mannitol (Man), pullulan (Pull), sucrose (Suc), sorbitol (Sor), trehalose (Tre), arginine (Arg), glycine (Gly), histidine (His). Error bars represent standard deviation of sample titers. Independent assays were repeated twice for each treatment, minimally, with multiple technical replicates per assay. Data were normalized to the mean viral titer of DENV-2 samples treated with HEPES buffer, represented as a stability value of (1) on the graph. Normalized data were used to determine statistical significance with One-Way ANOVA test Tukey’s HSD post hoc applied. Statistical significance is indicated as follows: *p < 0.05, **p < 0.01, ***p < 0.001, ****p < 0.0001.

The enhanced stability observed in combination formulations likely results from complementary mechanisms previously described for individual components. Carbohydrates may protect DENV-2 through preferential hydration and osmotic stabilization effects, while amino acids contribute through buffering effects or by binding to hydrophobic regions on envelope proteins. Previous research on H1N1 influenza subunit vaccines demonstrated that carbohydrate-amino acid combinations, including sucrose with glycine and trehalose with glycine, provided superior stability compared to individual components, though sucrose–mannitol combinations showed the lowest performance among tested combinations.^33^

When we organized formulations by amino acid component (Figure 5), histidine-containing combinations achieved the highest thermal protection compared to arginine and glycine formulations. Both arginine-carbohydrate and glycine–carbohydrate combination treatments exceeded HEPES control. However, histidine–carbohydrate combinations differed significantly from both arginine and glycine groups, indicating that histidine provides the most substantial stability enhancement across various carbohydrate partners.

Analysis of formulations grouped by carbohydrate component (Figure 6) revealed that all carbohydrate-containing treatments significantly outperformed the HEPES control, except for the sucrose and arginine combination. Carbohydrate combinations with histidine were consistently stable with the highest normal average values. This pattern suggests that carbohydrate selection has a limited impact on overall formulation stability within combination systems.

We conducted statistical analysis to evaluate the independent contribution of each excipient class to thermal stability by pooling all formulations containing the same component type, regardless of the partner compound. This approach assessed whether amino acids or carbohydrates served as the primary stability determinant in combination systems.

When all formulations were pooled by amino acid component (Figure 7A), histidine-containing combinations demonstrated superior thermal stability compared to arginine and glycine groups. Both arginine and glycine treatments exceeded the HEPES control performance with no statistically significant difference between these amino acids. However, histidine combinations differed significantly from both arginine and glycine groups, confirming that histidine provides the most substantial stability enhancement across all carbohydrate partners tested.

**Figure 7:**
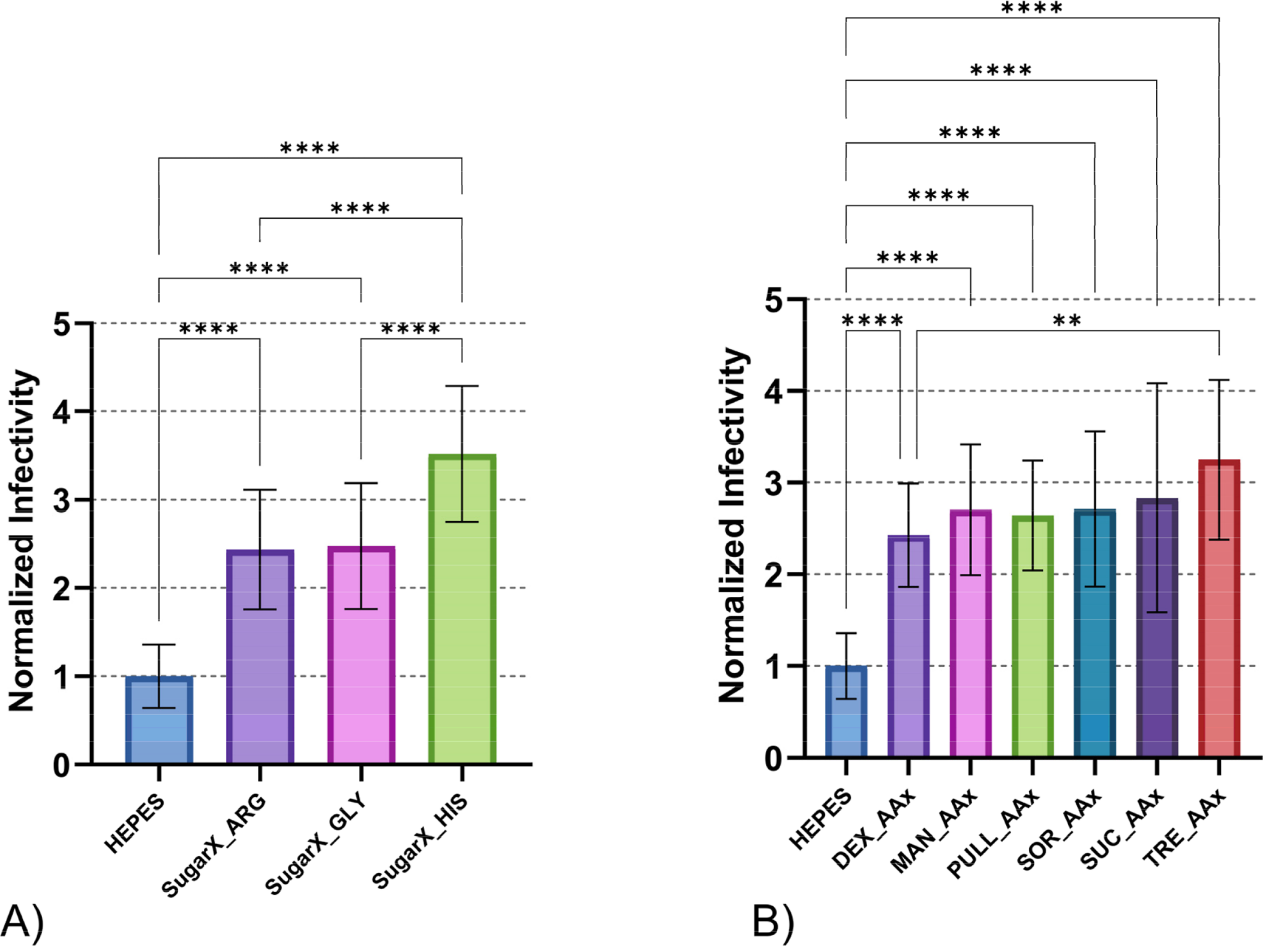
Thermal stability of DENV-2 after 24 h exposure at 21 °C. Data represents all formulations containing the same component regardless of partner compound: (A) formulations grouped by amino acid component; (B) formulations grouped by carbohydrate component. Abbreviations: dextran (Dex), mannitol (Man), pullulan (Pull), sucrose (Suc), sorbitol (Sor), trehalose (Tre), arginine (Arg), glycine (Gly), histidine (His). Error bars represent standard deviation of sample titers. Independent assays were repeated twice for each treatment, minimally, with multiple technical replicates per assay. Data were normalized to the mean viral titer of DENV-2 samples treated with HEPES buffer, represented as a stability value of (1) on the graph. Normalized data were used to determine statistical significance with One-Way ANOVA test Tukey’s HSD post hoc applied. Statistical significance is indicated as follows: *p < 0.05, **p < 0.01, ***p < 0.001, ****p < 0.0001.

Pooled analysis by carbohydrate component (Figure 7B) revealed that all carbohydrate-containing treatments significantly outperformed the HEPES control. Post hoc analysis showed minimal differences between carbohydrate groups, with only dextran and trehalose demonstrating statistically significant differences. This finding indicates that carbohydrate identity has a limited impact on overall thermal stability when combined with amino acids.

The pooled analysis confirms that amino acid selection, particularly histidine inclusion, exerts greater influence on thermal stability than carbohydrate choice in dual-excipient formulations. The consistent performance across different carbohydrates demonstrates that the amino acid component serves as the primary stability determinant, suggesting that histidine-based formulations would be optimal regardless of carbohydrate partner selection.

### Ionic Liquid-Based Formulations

Biomolecule stabilization using deep eutectic solvents (DESs) represents an emerging strategy in biotechnology.^40–42^ These systems, similar to excipients, are organic salts with relatively high viscosity whose interactions with water and proteins are dominated by hydrogen bonding.^43,44^ Choline-based ILs, which combine the cationic essential nutrient choline with various biocompatible anions, have gained significant attention for enhancing protein stabilization.^45^ Choline is attractive as a cation for biocompatible compounds due to its biological origin and structural features that promote low toxicity, including short alkyl chains and a hydroxyl group. Importantly, choline and the selected anions (chloride, acetate, dihydrogen phosphate) are all GRAS by the FDA and have established histories of use in pharmaceutical formulations, making them suitable candidates for vaccine applications that require stringent safety profile.^46^

Hallett and colleagues demonstrated that choline-based IL systems combined with carbohydrates and amino acids, can preserve the structural integrity and conformational stability of proteins, including therapeutic antibodies.^24,25^ Their research showed that formulations containing choline dihydrogen phosphate, trehalose, and arginine effectively stabilized complex protein structures by modulating protein–solvent interactions through preferential binding mechanisms and conformational landscape engineering. Additionally, choline-based ILs have demonstrated suitability for protein extraction and purification without causing degradation or denaturation effects.^47,48^

Specific DES formulations have shown remarkable promise for vaccine applications. A trehalose-glycerol DES system has been shown to effectively stabilize influenza hemagglutinin-displaying virus-like particles.^49^ Additionally, Mitragotri and co-workers explored choline-based ILs as vaccine adjuvants, initially demonstrating that choline and lactic acid formulations can enhance immune infiltration at injection sites and produce potent immune responses,^50^ and subsequently developed choline and sorbic acid as an IL adjuvant that generates both cellular and humoral immune responses against multiple antigens.^51^ These findings suggest that choline-based IL formulations could provide a tunable platform for stabilizing labile biological entities, including vaccine antigens.

Based on these findings, we hypothesized that IL formulations serve as stabilizing agents for flavivirus vaccines. To test this hypothesis, we evaluated four choline-based ILs: choline chloride (ChCl), choline acetate (ChOAc), choline acetyl chloride (ChAcetylCl), and choline dihydrogen phosphate (ChDHP), each combined with the most promising carbohydrates and amino acids identified in our earlier studies (Figures 2 and 8). Initial screening revealed that ChAcetylCl and ChDHP provided no stabilizing effect for DENV-2 and were therefore excluded from further analysis (data not shown). The lack of stabilization by ChAcetylCl can be attributed to the hydrolytic instability of acetyl chloride groups in aqueous environments, which rapidly convert to acetic acid and HCl, creating acidic conditions detrimental to viral envelope integrity. Similarly, ChDHP may have interfered with the optimal pH buffering provided by the HEPES system, as the protic DHP anion could disrupt the physiological pH conditions essential for flavivirus stability. While ChCl and ChOAc formulations did provide measurable thermal protection, particularly when combined with sucrose and histidine, their stabilizing effects were less pronounced compared to conventional carbohydrate-amino acid combinations. This suggests that the stabilization mechanisms governing intact flavivirus vaccine-like models differ from those observed in isolated protein systems, potentially due to the complex multicomponent architecture of viral particles, including envelope proteins, nucleic acids, and lipid membranes.

**Figure 8:**
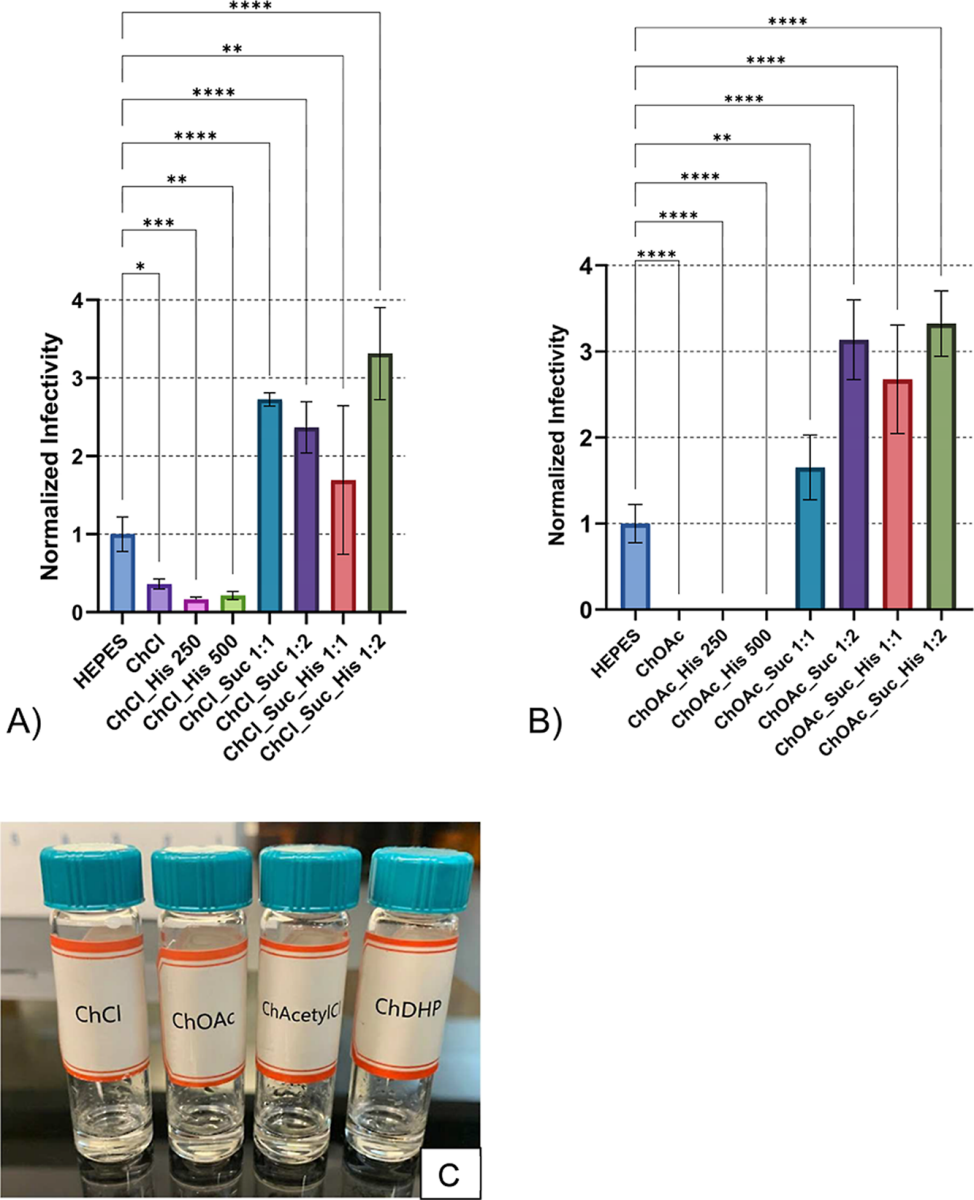
Thermal stability of DENV-2 after 24 h incubation at 21 °C for choline-based formulations combined with carbohydrate and amino acid excipients. (A) Choline chloride (ChCl) formulations with varying concentrations of sucrose and histidine; (B) choline acetate (ChOAc) formulations with varying concentrations of sucrose and histidine. Error bars represent standard deviation of sample titers. Independent assays were repeated twice for each treatment, minimally, with multiple technical replicates per assay. Data were normalized to the mean viral titer of DENV-2 samples treated with HEPES buffer, represented as a stability value of (1) on the graph. Normalized data were used to determine statistical significance with One-Way ANOVA test Tukey’s HSD post hoc applied. Statistical significance is indicated as follows: *p < 0.05, **p < 0.01, ***p < 0.001, ****p < 0.0001. (C) Representative images of final formulations containing choline salts with sucrose and histidine in glass vials.

Figure 8 depicts the stability results for the remaining DES formulations. ChOAc-based systems demonstrated superior stability compared to ChCl formulations when looking at average normalized values. Importantly, formulations combining IL with sucrose, or with both sucrose and histidine, showed statistically significant improvements in viral stability relative to the HEPES control (Figure 8). However, treatments containing ChCl-alone, ChOAc-alone, or their relative histidine-only combinations failed to improve stability beyond the control level. The stabilizing effects observed with ChCl and ChOAc likely arise from the ability of chloride and acetate counterions to form stabilizing interactions with viral proteins through H-bonding and electrostatic mechanisms, complementing the glass-forming properties of the choline cation. While the DES formulations evaluated in this study provided enhanced thermal stability compared to buffer alone, they did not surpass the stabilization achieved by carbohydrate-amino acid combinations (vide supra).

The observed decrease in viral stability with increasing IL concentrations can be attributed to osmotic stress and ionic strength effects on the flavivirus envelope. As IL concentrations increase, the osmotic pressure of the formulation rises, creating conditions that can disrupt the lipid bilayer integrity of the viral envelope through dehydration and membrane distortion. Additionally, elevated ionic strength may interfere with the electrostatic interactions that stabilize viral capsid proteins and envelope glycoproteins, potentially leading to conformational changes that reduce infectivity. These effects are particularly pronounced in enveloped viruses like DENV-2, YFV-17D, and JEV, where membrane stability is critical for maintaining viral viability. The concentration-dependent decline in stability suggests an optimal ionic strength threshold exists, beyond which the disruptive osmotic effects outweigh any potential stabilizing benefits of the DES formulation.

### Cross-Viral Validation: Extension to YFV-17D and JEV Models

To assess the broad applicability of our stabilization approach across the flaviviruses, we evaluated the most effective formulations identified in the DENV-2 studies against two additional flavivirus vaccine models: YFV-17D and JEV. This cross-viral validation was essential to determine whether the stabilization mechanisms observed for DENV-2 represented a generalizable strategy applicable to structurally related flaviviruses.

The formulations selected for cross-viral testing included those combining carbohydrates (trehalose and sucrose) with histidine, which had demonstrated the greatest thermal protection for DENV-2 in our previous experiments. Figure 9 presents the comparative thermal stability profiles for all three vaccine models across the selected formulation treatments. Each treatment group was evaluated against two controls: desiccated virus without buffer supplementation, and virus preserved in HEPES buffer alone.

**Figure 9:**
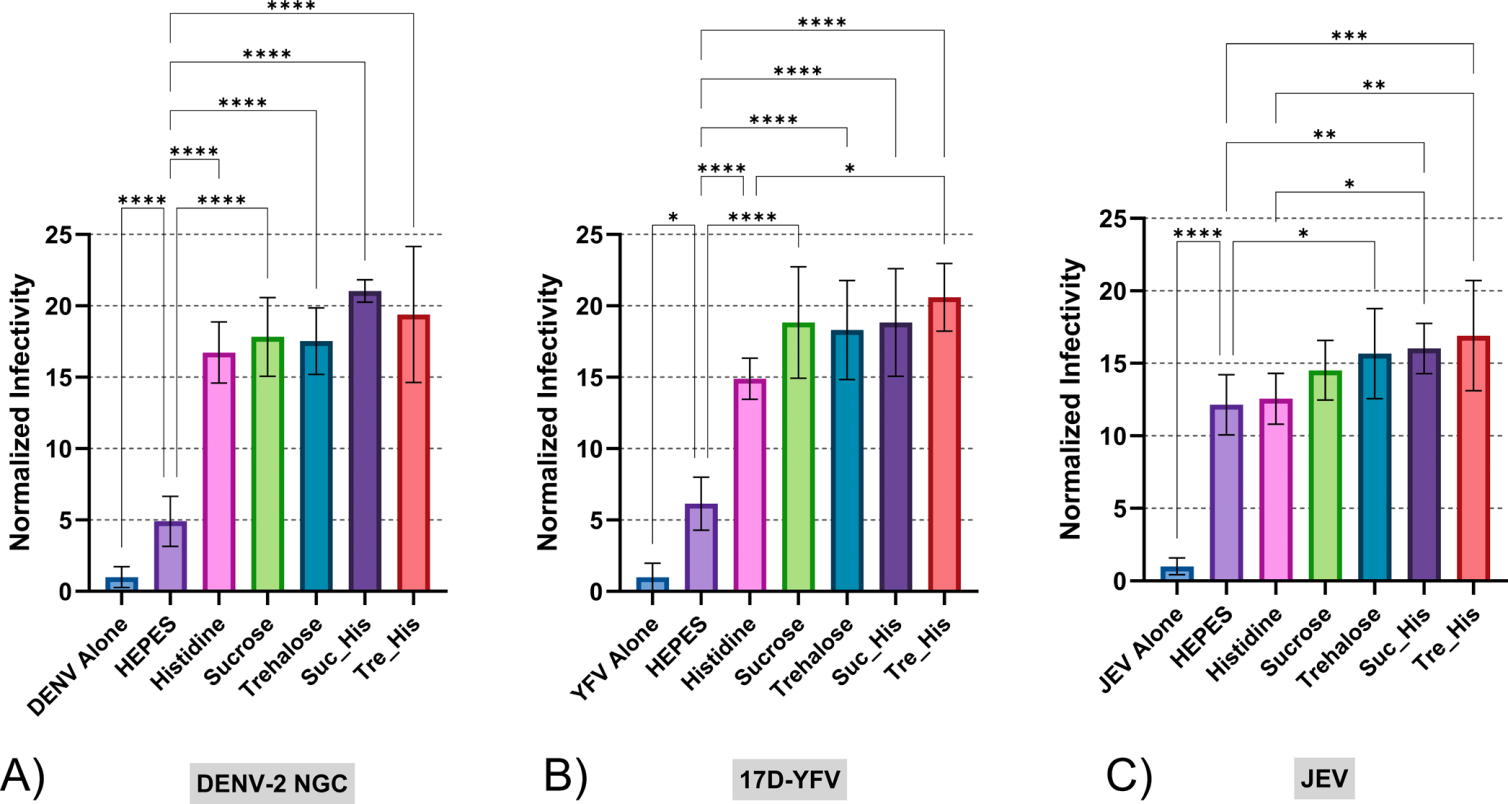
Thermal stability of (A) DENV-2 NGC, (B) 17D-YFV, and (C) JEV after 24 h incubation at 21 °C. Abbreviations: dextran (Dex), mannitol (Man), pullulan (Pull), sucrose (Suc), sorbitol (Sor), trehalose (Tre), arginine (Arg), glycine (Gly), histidine (His). Error bars represent standard deviation of sample titers. Independent assays were repeated twice, minimally, with multiple technical replicates per assay. The resulting virus titers were normalized to the total average titer of each respective virus with No HEPES treatment, represented as a baseline for comparison, value of (1) in the graph, within each virus group. A One-Way ANOVA was used to compare treatments shown within each virus group, with Tukey’s HSD post hoc applied. Statistical significance is indicated as follows: *p < 0.05, **p < 0.01, ***p < 0.001, ****p < 0.0001.

Statistical analysis revealed that all tested formulations produced significant improvements in viral stability compared to both control conditions across all three vaccine models (*p* < 0.05). This consistent performance across phylogenetically related but antigenically distinct flaviviruses supports the hypothesis that our carbohydrate-amino acid formulations stabilize conserved structural features common to the Flaviviridae family, rather than virus-specific epitopes. The magnitude of stabilization varied among the three viruses, with DENV-2 showing the greatest response to formulation treatment, followed by YFV-17D and JEV.

Notably, the JEV vaccine model exhibited a greater baseline response to pH buffer composition. While DENV-2 and YFV showed stability differences between unbuffered controls and HEPES alone, JEV demonstrated significantly enhanced thermal stability in HEPES compared to the unbuffered control by nearly 1 log. This finding suggests that the JEV chimera virus may possess inherent sensitivity to pH maintenance during desiccation and storage, independent of excipient-mediated stabilization. The mechanistic basis for this virus-specific buffer response warrants further investigation, as it may reflect differences in surface charge distribution or conformational flexibility among flavivirus envelope proteins.

Despite the differential buffer responses observed, the carbohydrate-amino acid formulations consistently outperformed both control conditions for all three viruses, demonstrating the robust and generalizable nature of this stabilization approach. These results show that our formulation strategy can be effectively extended beyond DENV-2 to address thermal stability challenges for multiple flavivirus vaccines, supporting the development of thermostable vaccine formulations for deployment where YFV, JEV, and DENV cocirculate and cold chain infrastructure remains limited.

## Conclusion

This study evaluated the effects of several excipients on the thermal stability of three flavivirus vaccine models (DENV-2, YFV-17D, and JEV) following a 24 h incubation period at ambient temperature (21 °C). The results identified multiple excipient candidates capable of maintaining vaccine stability under accelerated stress conditions. Among the formulations tested, the combination of trehalose and histidine demonstrated the highest stabilizing effects across all three vaccine models. Further development of this excipient combination could facilitate the distribution of live-attenuated flavivirus vaccines to populations in regions where maintaining cold chain logistics systems is challenging.

This work also explored the potential of choline-based deep eutectic solvents (DES) as novel stabilizing agents for flavivirus vaccines. While formulations containing choline chloride and choline acetate combined with sucrose and histidine provided measurable protection, their stabilizing effects did not surpass those achieved by carbohydrate-amino acid combinations. Future work should emphasize stabilizers that better protect exposed hydrophobic envelope regions and preserve lipid-bilayer integrity.

The inclusion of HEPES as a buffering system proved essential for maintaining an optimal pH environment for flavivirus vaccine stability. While previous studies have employed phosphate-buffered saline (PBS) for stabilization research, comparative studies evaluating HEPES against PBS may further elucidate the superior buffering capacity observed in this work. An explanation of the substantial difference in stability seen between HEPES and phosphate buffering systems could be the differential solubility of monocation versus dication versions of the phosphate ion. During a drying (or freezing) process, less soluble disodium phosphate will precipitate out first, causing a large change in pH.^31^ This result alone suggests that phosphate based pH buffer systems are not preferable for biological drying or freezing applications, especially for pH sensitive products like live-attenuated flavivirus vaccines.

This work suggests several avenues for future investigation. First, evaluating the stability of these excipient formulations following vaccine reconstitution would provide critical information for practical implementation. Second, extending the accelerated stability studies to higher temperatures (e.g., 37 °C) and longer durations (one week to one month) would better emulate real-world storage scenarios. Finally, mechanistic studies elucidating how these excipients interact with solvent-exposed surface residues of envelope proteins and lipid bilayers would provide fundamental insights to guide the rational design of next-generation thermostable vaccine formulations.^31^ These formulations show particular promise for short-term ambient transport in resource-limited settings where cold-chain infrastructure is unreliable, potentially reducing the 50% vaccine waste currently attributed to cold-chain failures.
